# Complex biomechanical properties of non-augmented and augmented pedicle screws in human vertebrae with reduced bone density

**DOI:** 10.1186/s12891-020-3158-z

**Published:** 2020-03-06

**Authors:** Martin Schulze, Oliver Riesenbeck, Thomas Vordemvenne, Michael J. Raschke, Julia Evers, René Hartensuer, Dominic Gehweiler

**Affiliations:** 1 University Hospital Münster, Department of Trauma, Hand and Reconstructive Surgery, Albert-Schweitzer-Campus 1, 48149 Münster, Germany; 2 University Hospital Münster, Department of General Orthopaedics and Tumor Orthopaedics, Albert-Schweitzer-Campus 1, 48149 Münster, Germany; 3 Evangelical Hospital Bethel GmbH, Department of Trauma Surgery and Orthopaedics, Bielefeld, Germany; 4 AO Research Institute Davos, Davos, Switzerland

**Keywords:** Physiologically related setup, Lumbar vertebrae, Cyclic loading, Pedicle screws, Biomechanics, Reduced BMD

## Abstract

**Background:**

In osteoporotic bone, the quality of the bone-to-implant interface is decreased, which may lead to early implant failure. Screw anchorage can be improved by augmentation. This effect is mainly investigated with a pull-out test. To our knowledge, the effect of cement augmentation in an in vivo physiological setup focusing on screw movement has not been investigated to date. The aim of this work was to investigate and compare augmented and native screw behavior in a physiologically related setup.

**Methods:**

Twelve fresh-frozen human lumbar vertebrae were divided into two groups. Each vertebra was bilaterally instrumented with either non-augmented or augmented pedicle screw systems and loaded in a recently developed test setup that provided cyclic conditions comparable to a physiological gait. The cyclic loading should test the primary implant stability, comparable to the postoperative period of two months in a worst-case scenario in the absence of osseous remodeling. Screws were tracked optically, and screw movement and failure patterns were observed.

**Results:**

Mutual influence between the left and right sides resulted in a successive, rather than simultaneous, failure. Augmentation of the screws in vertebrae with poor bone quality reduced screw subsidence and thus improved the rigidity of the screw-to-implant interface by up to six-fold. The non-augmented condition was significantly related to early screw failure.

**Conclusions:**

Pedicle screw system failure involves a complex bilateral-coupled mechanism. The cyclic loading based on physiological conditions during walking has allowed the postoperative conditions and clinical failure mechanisms to be simulated in vitro and clarified. Future implant systems should be investigated with a physiologically related setup.

## Background

Instrumented stabilization of the spinal column depends on adequate anchoring of the implants in the bone, which is essentially based on the quality of the bone-to-implant interface [1].

The altered metabolism in osteoporosis can delay fusion due to prolonged time until callus formation occurs and is combined with reduced implant anchorage, resulting in reduced secondary stability [2–4].

Clinically, this situation and its potential risk of increased failure may be improved by cement augmentation of screws. According to several pull-out biomechanical tests, screw anchorage is improved by 160% and up to twice the pull-out force of the non-augmented pedicle screw for each osteoporotic grade [5, 6].

However, as the physiological loading of the spine is very complex, it can serve as a template for an experimental analysis of the bone-to-implant interface. Several studies investigated unilateral cyclic loading under varying conditions while the vertebra was rigidly fixed [7–9]. Implant loads of max. 300 N under compression and bending moments from 3.0–7.5 Nm were measured in vivo [10], which differed from the conditions employed in many experiments.

As reported elsewhere, we developed a method to apply quasi-physiological loading and detect screw movement [11]. To our knowledge, the effect of cement augmentation in an in vivo physiological setup focusing on the observed screw movement has not been investigated to date.

For these reasons, the aim of this work was to investigate and compare augmented and native screw behavior in a physiologically related setup.

## Methods

### Grouping and sample preparation

Twelve fresh-frozen human lumbar vertebrae from L1–L4 with a mean age of 72.2 ± 9.9 (range from 59 to 94) years were tested. All donors originated from the anatomical department of the University of Muenster and had given written consent to dedicate their bodies to medical education and biomedical research. The bone mineral density (BMD) was determined using quantitative computer tomography (SOMATOM Sensation, Siemens Medical Solutions, Erlangen, Germany). Pedicle height and width were measured at the thinnest part of the pedicle in craniocaudal respectively mediolateral direction of the pedicle in the CT. The pedicle angle was determined as the angle between the pedicle and the median plane, also on CT. The samples were divided into two groups to ensure an equal BMD distribution. Each vertebra was instrumented by a senior surgeon with two cannulated pedicle screws with dimensions of 6 × 45 mm (DSS, Paradigm Spine, Wurmlingen, Germany): group 1, non-augmented; group 2, augmented. Each screw in group 2 was augmented with 2.5–3 ml polymethyl methacrylate cement (PMMA - Vertecem®, DePuy Synthes, Zuchwil, Switzerland). Implantation and augmentation were performed under radiographic control (POWERMOBIL®, Siemens Medical Solutions, Erlangen, Germany).

### Test setup and parameters

**Fig. 1:**
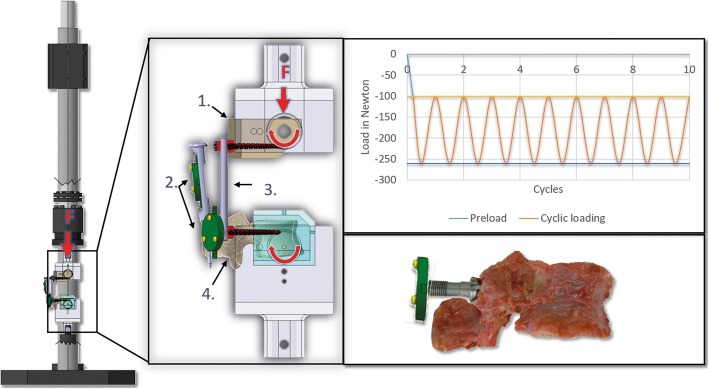
Testing setup. The cranial pedicle screws are anchored in organic thermoplastic polymer (PEEK) according to (ASTM) F-1717-04 (1). The rigid body (2) is rigidly fixed on the caudal screw and the spinous process for active motion tracking. The cranial and one caudal pedicle screw are rigidly connected with a longitudinal rod (3). The caudal screws are placed in the mounted vertebra (4). Both (1) and (4) can be tilted in ventral and dorsal directions by means of the pivot axis bearings of the setup

The setup used a modified standard of the American Society of Materials and Testing (ASTM) F-1717-04 [12] for preclinical evaluations of pedicle screw systems and was described in detail previously (Fig. 1) [11].

A total of 60,000 cycles were applied with a servohydraulic testing machine (Instron-8874, Norwood, MA, USA). The number of cycles was derived by assuming postoperative bony bridging after 2 months and an average activity of 1000 steps/day during convalescence [10, 13, 14]. The cyclical loading frequency was set to 1.83 Hz, corresponding to a physiological cadence of 110 steps/min [15–17].

The cyclic test protocol started with a preloading of 100 N, which was set as the lower limit of cyclic loading. An additional loading of 160 N was set as the upper limit.

The lever arm remained constant at 45 mm, corresponding to a sagittal torque of 5.85 Nm for each screw. All selected test parameters corresponded to in vivo measurements [10, 15]. Testing was terminated at a height reduction of 10 mm under preload and 15 mm under preload plus cyclic loading, which was defined as abort criterion.

### Screw characteristics and movement tracking

Each screw movement was recorded relative to the vertebra with 50 Hz using an optical measuring system (NDI Optotrak-Certus, Waterloo, Ontario, Canada). Infrared markers were mounted to the head shank (screw head) to ensure that the polyaxial mechanism of the system did not falsify the measurement of the screw. The tip was referenced by a pivot algorithm, transforming the base of the screw head into the tip. The reference was attached to the spinous process.

The displacement evaluation for the screws was performed in MATLAB R2013b (The MathWorks Inc., Natick, MA, USA).

The positions of the screw head and tip were recorded in unloaded condition (condition 0) prior to load application, at the lower limit load (condition 1) and at the upper limit load condition (condition 2). A change in these position values was defined as displacement, which was calculated for the beginning and the termination of each testing.

Every 1000 cycles in the progress of displacement were compared and defined as progress of displacement (PD).

Furthermore, the movement between condition 1 and condition 2 was detected for each cycle. The positions of the screw head and tip were calculated for each condition and each cycle. The distance between the position in condition 1 and in condition 2 defined the resulting movement.

Diagrams were generated for each screw and parameter, allowing qualitative analysis of screw movement over the number of cycles.

In addition, each cycle was analyzed for movement patterns in order to assign them to one of the following movement categories: pure translation, pure rotation, combined rotation and translation, the so-called toggling, and undefined movement pattern.

Category 1: A translational movement was detected when a simultaneous movement of the screw head and screw tip in craniocaudal direction was measured.

Category 2: A rotational movement was detected if an opposite movement occurred in craniocaudal direction for the screw-head and screw-tip, i.e. a simultaneous cranial movement of the head and a caudal movement of the tip, which results in a rotation with a pivot point in or near to the pedicle.

Category 3: A combined rotation and translation, the so-called toggling, was defined as rotational movement (see above) with a temporary change of direction of the tip movement (translation). Therefore, the algorithm had to detect two toggle points at the tip of the screw and only one toggle point for the head.

Category 4: Undefined movement pattern, which was defined for all movements that could not be assigned to one of the categories 1 to 3.

The morphological parameters of the pedicle were examined for possible correlations with the displacement parameters of the screws.

The results were not distributed normally and are hence displayed as median plus first/third quartile. The Wilcoxon-signed-rank test was performed using SPSS Statistics 24 (IBM, Armonk, NY, USA) to detect significant differences in the movement of the screw head and tip. The Mann-Whitney-U test was used to detect differences between groups. The significance level was set to *p* = 0.05. The Spearman correlation coefficient was calculated between screw movement and morphological data.

## Results

The specimens’ calcium hydroxyapatite content was 102.8 (84.1/104.4) (52.8–109.8) mg/ml, indicating osteoporosis or osteopenia [18]. The median BMD values for group 1 and 2 were not significantly different (102.8 (90.9/108.7) (63.5–109.8) mg/ml respectively 102.8 (63.5/103.0) (52.8–108.7) mg/ml).

### Subsidence under initial load application

**Fig. 2:**
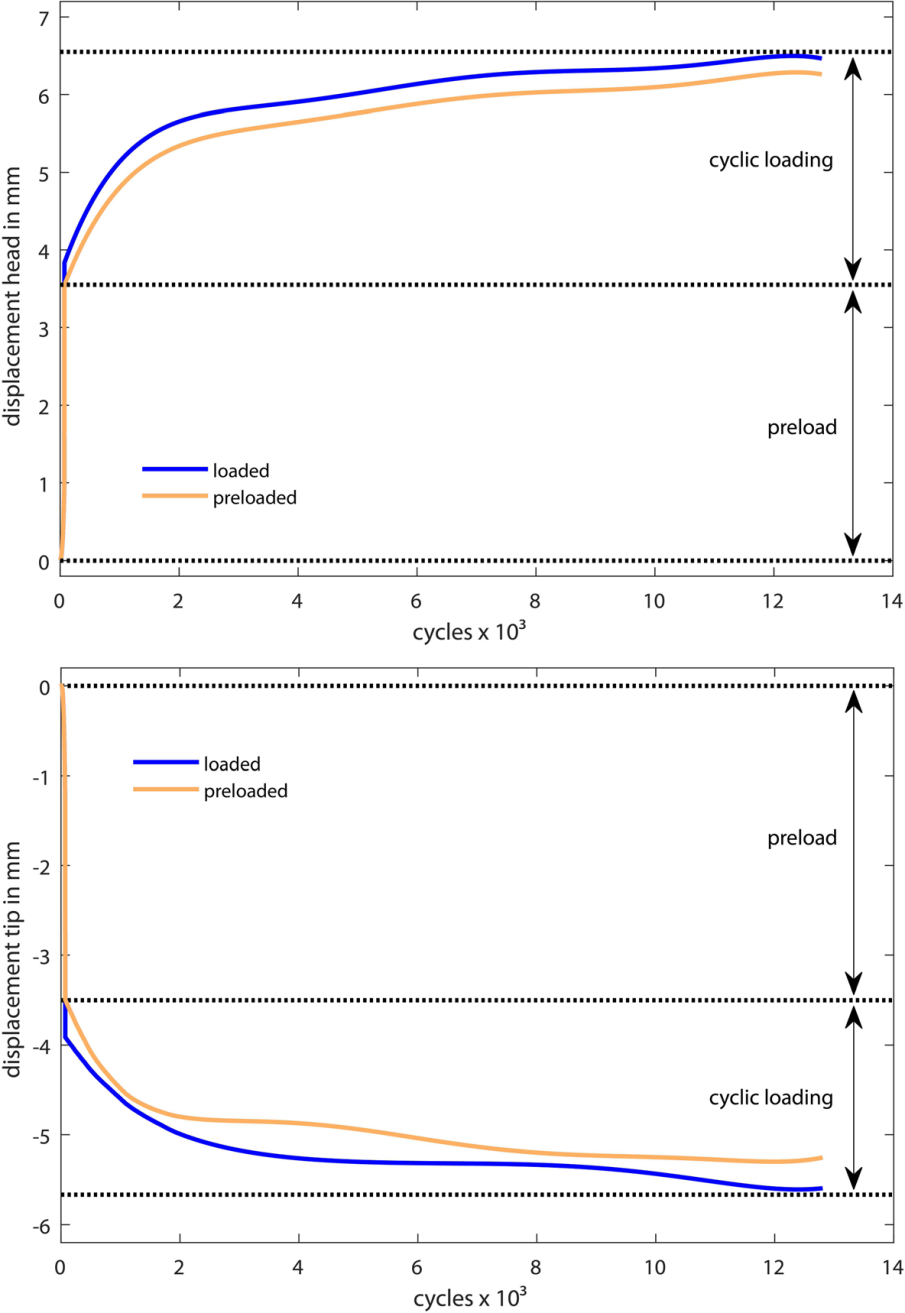
Representative screw displacement of a non-augmented vertebra under preload (condition 1) and cyclical load (condition 2) over 13,000 cycles until the machine limits are reached. Top: The screw head moved in cranial direction (positive sign). Bottom: The screw tip moved in caudal direction (negative sign); preloaded; loaded = preload + cyclic load

After initial load application (condition 1), all screw tips moved caudally (negative values) and all screw heads moved cranially (positive values) except for one specimen in group 1 (Fig. 2). There, a co-directional movement of head and tip was detected. This vertebra showed a BMD with 103 mg/ml and the smallest pedicles with 6 mm in height and width.

The movement of the head was less significant in group 2 than in group 1 (*p* = 0.043). Movements of the augmented screw tips was also smaller but not significantly different from that of the non-augmented tips (*p* = 0.193).

However, a significant difference in stiffness was detected for the screw head, and there was higher stiffness in group 2.

There was a significantly smaller deflection of the screw in augmented group 2 after the initial load application. Detailed values are summarized in Table 1.

**Table 1:** Results of the motion parameters

	Group 1 (non-augmented)		Group 2 (augmented)	
Parameter	Screw head	Screw tip	Screw head	Screw tip
**Pdisp**	0.5 (0.3/1.3)*	−0.7 (− 0.6/−1.0)	0.3 (0.3/0.4)*	− 0.6 (− 0.3/− 0.8)
**Pdefl**	1.4° (1.3°/3.0°)*		1.2° (1.1°/1.4°)*	
**Pstiff**	216 (76/332)	140 (105/162)*	295 (285/385)	159 (129/297)*
**Cdisp**	1.7 (0.5/2.9)*	−1.2 (−0.5/− 1.9)*	0.3 (0.2/0.3)*	−0.1(− 0.1/− 0.2)*
**DEFL**	7.0° (2.2°/9.6°)**		1.9° (1.6°/2.0°)**	
**STIFF**	80 (63/316)**	122 (83/277)*	475 (417/602)**	298 (263/340)*
**Cmov start**	0.6 (0.5/0.6)	0.9 (0.8/1.2)*	0.5 (0.3/0.5)	0.7 (0.6/0.9)*
**Cmov end**	0.4 (0.3/0.4)	0.7 (0.5/0.8)	0.6 (0.3/0.6)	0.6 (0.5/0.7)
**PDmax**	0.14 (0.07/0.42)*	0.17 (0.11/0.45)*	0.02 (0.01/0.02)*	0.05 (0.03/0.05)*

Subsidence under preload (Pdisp, n = 12) and its deflection (Pdefl, n = 12) and stiffness (Pstiff, n = 12), subsidence under cyclic loading (Cdisp, n = 10), total deflection (DEFL, n = 10), total stiffness (STIFF, n = 10), screw loosening (Cmov, n = 10), maximum progress of displacement (PDmax, n = 10). Stiffness is in N/mm, and all other values are in mm (Q25/Q75). Significantly different * (p < 0.05), ** (p < 0.01) between group 1 (non-augmented) and group 2 (augmented). The outliers were eliminated from cyclic analysis

### Displacement evaluation under cyclic loading

**Fig. 3:**
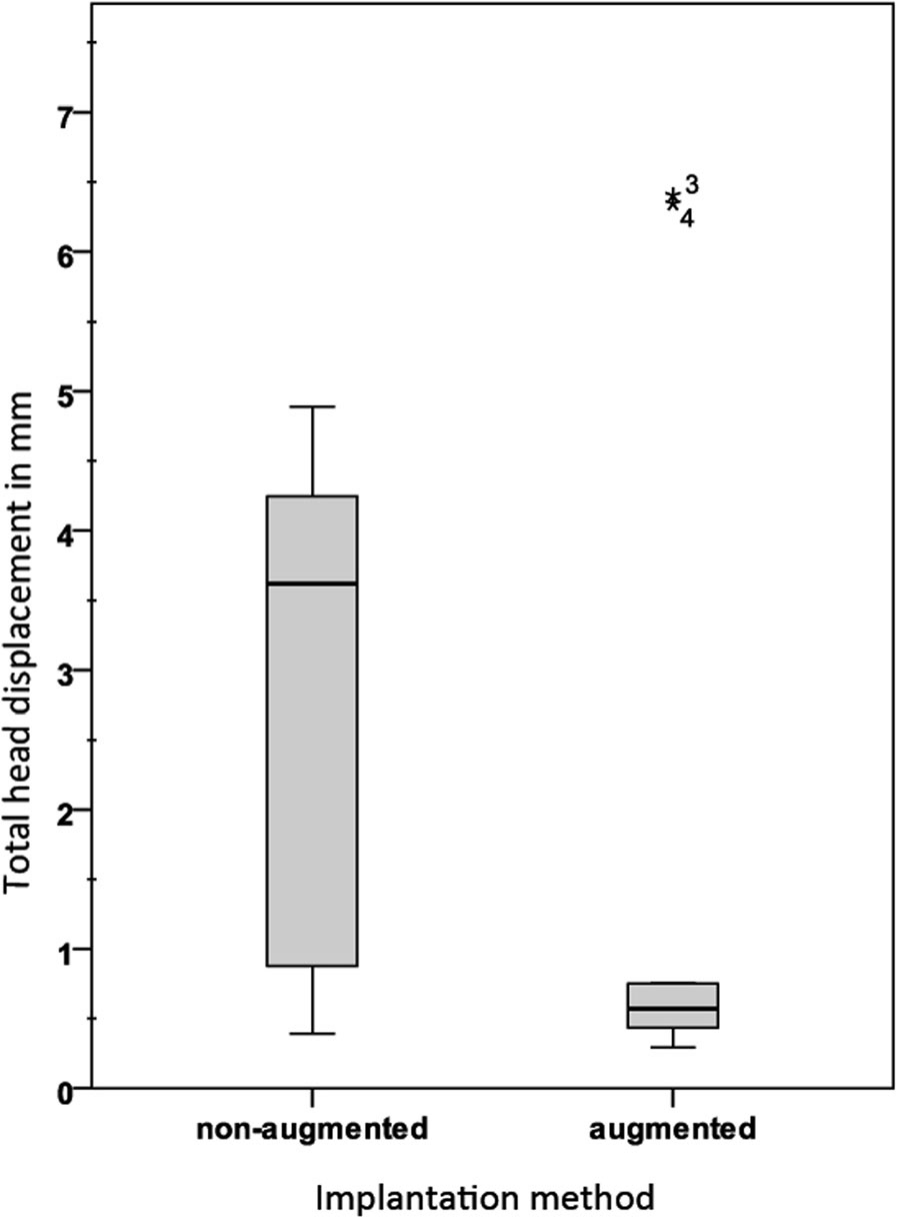
Total displacement under preload and under cyclic loading for screw head. Numbers 3 and 4 mark the outlier samples of group 2

**Fig. 4:**
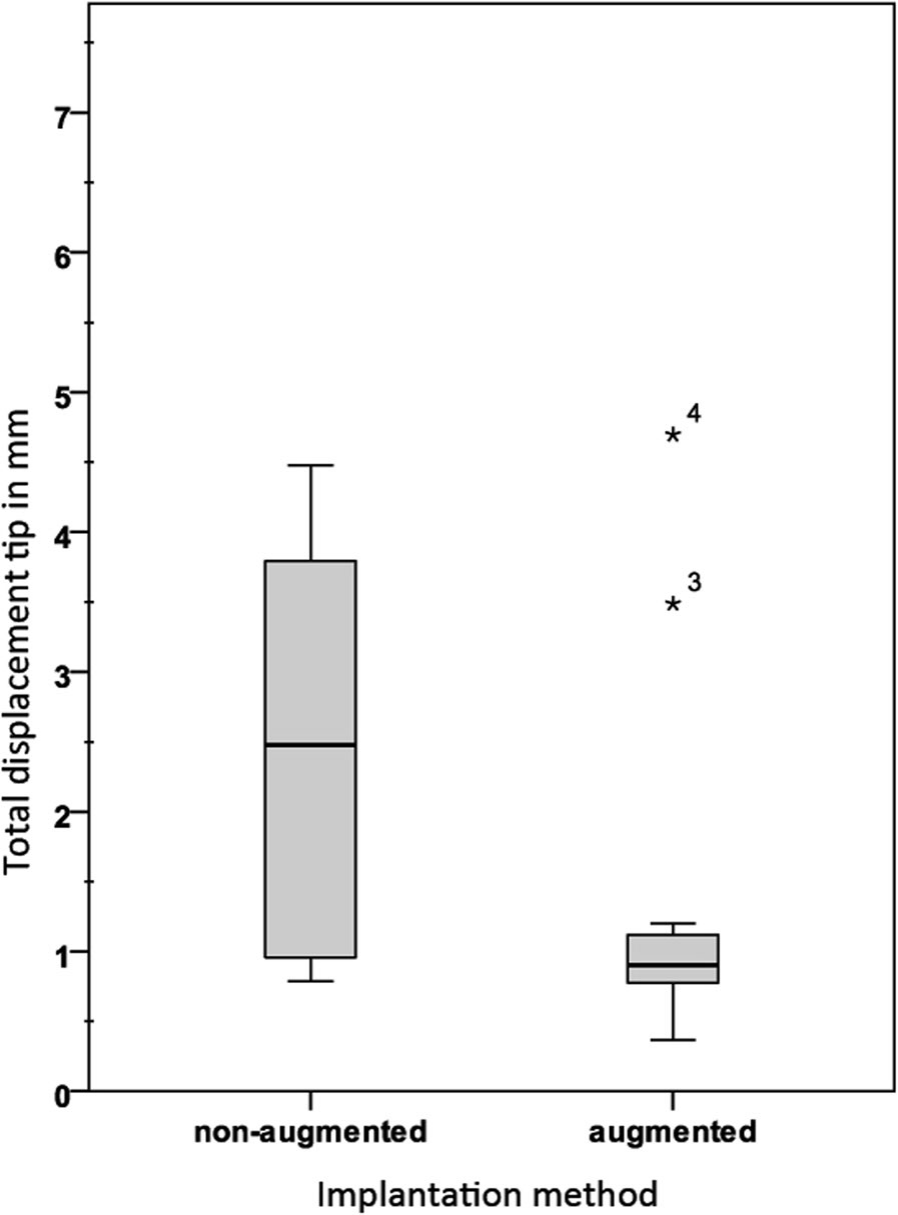
Total displacement under preload and under cyclic loading for screw tip. Numbers 3 and 4 mark the outlier samples of the augmented group 2

In the subsequent cyclic loading, displacement was significantly lower in group 2 at the head (*p* = 0.011) and at the tip (*p* = 0.000) compared to the values in group 1 (Fig. 3, Fig. 4).

The total deflection for head and tip in group 1 was more than 3 times greater than in group 2 (*p* = 0.007).

Stiffness in group 2 increased by 6-fold for the head (*p* = 0.005) and 2.5-fold for the tip (*p* = 0.015) compared to that in group 1. Detailed results are summarized in Table 1.

### Failure under cyclic loading

In group 2, all specimens reached the scheduled 60,000 cycles as described above. However, one specimen of the augmented group showed clear outliers and revealed a structural damage at the pedicles. Therefore, this sample had to be excluded from further statistical analysis after initial loading.

In group 1, only two vertebrae reached the total cycles. The other samples in group 1 reached abort criterion after 6500/13,000/35,000 cycles.

If early failure of the screws is considered as the endpoint, Fischer’s exact test shows a significant relationship between the frequency of early failure and the non-augmented group 1 (*p* = 0.011).

However, a macroscopically fractured pedicle was only found in the sample that stopped after 6500 cycles. Consequently, this sample had to be excluded as well from further statistical analysis.

**Fig. 5:**
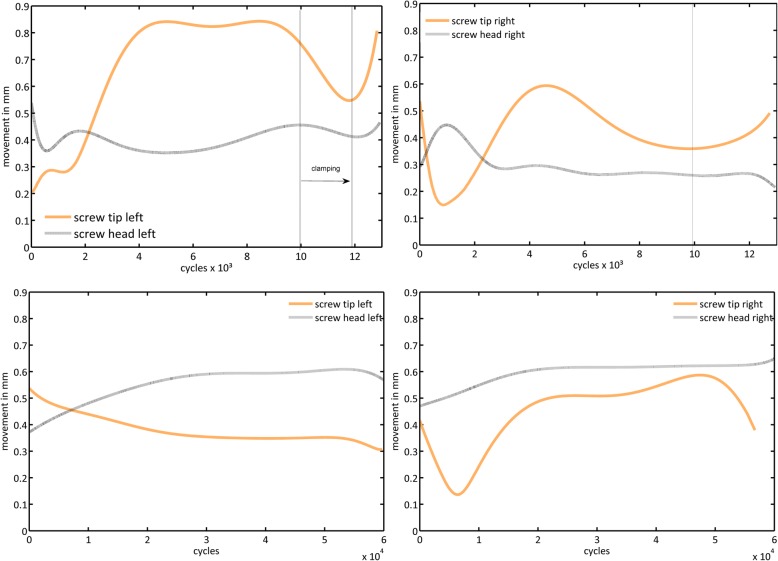
Side-by-side comparison of a representative screw movement in non-augmented (top) and augmented (bottom) samples with subsequent load change. Typical windshield wiper effect of both screw tips in the non-augmented sample until approx. 4000 cycles, when the screw tip loosens (left, right top); beyond 4000 cycles, a typical effect of increased load transfer by screw clamping (until dashed line) occurs within the right pedicle (right top); after approx. 10,000 cycles, the right tip increases again and a load transfer to the left screw follows until 12,000 cycles. Subsequently, the loosening increases for both sides until machine abort criterion are reached - displayed as absolute values

Movement in group 1 of the first and final cycles of the tip was approximately 1.5-fold greater than the head movement of the screw, thus presenting the typical windshield wiper effect (Table 1). Movement was not constant over time. The change in movement is presented in Fig. 5.

**Fig. 6:**
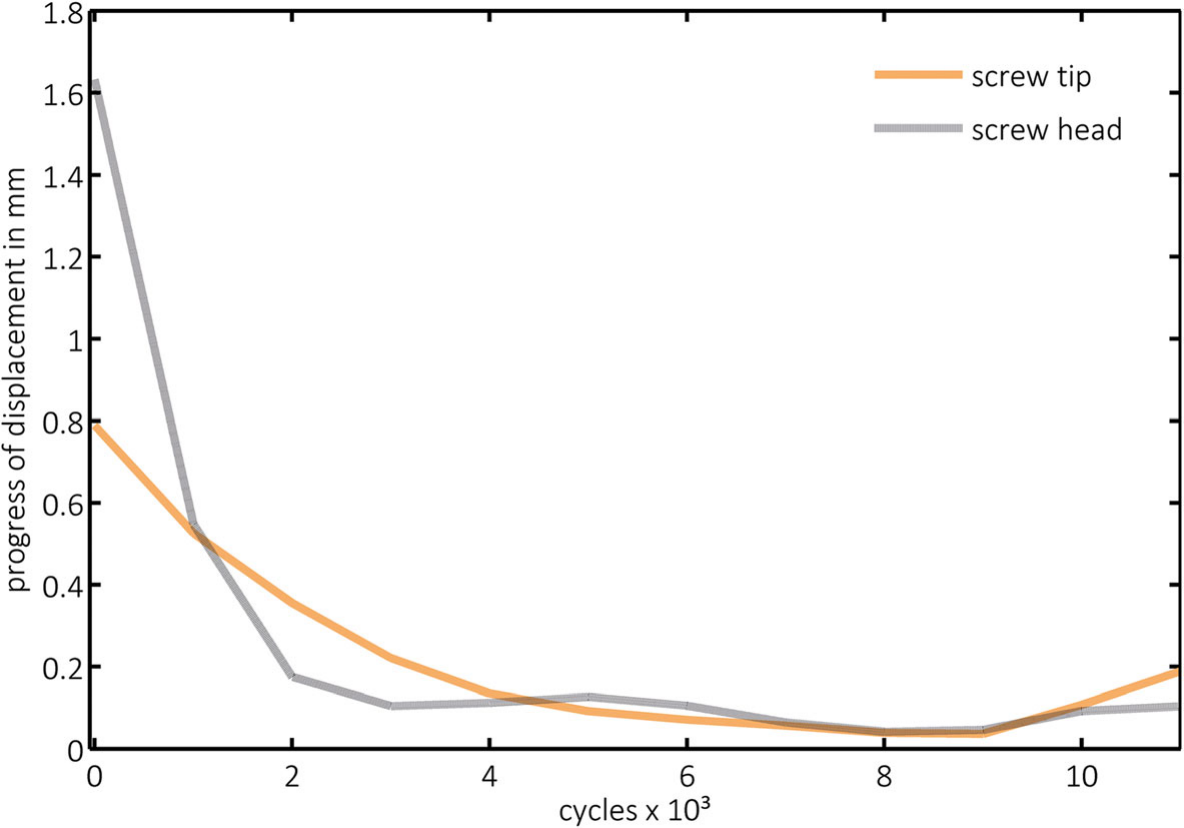
Exemplary progress of the screw displacement in group 1 (non-augmented). Progress of displacement (PD) and the corresponding number of cycles were calculated. Before reaching the machine limits, the progress increases

**Fig. 7:**
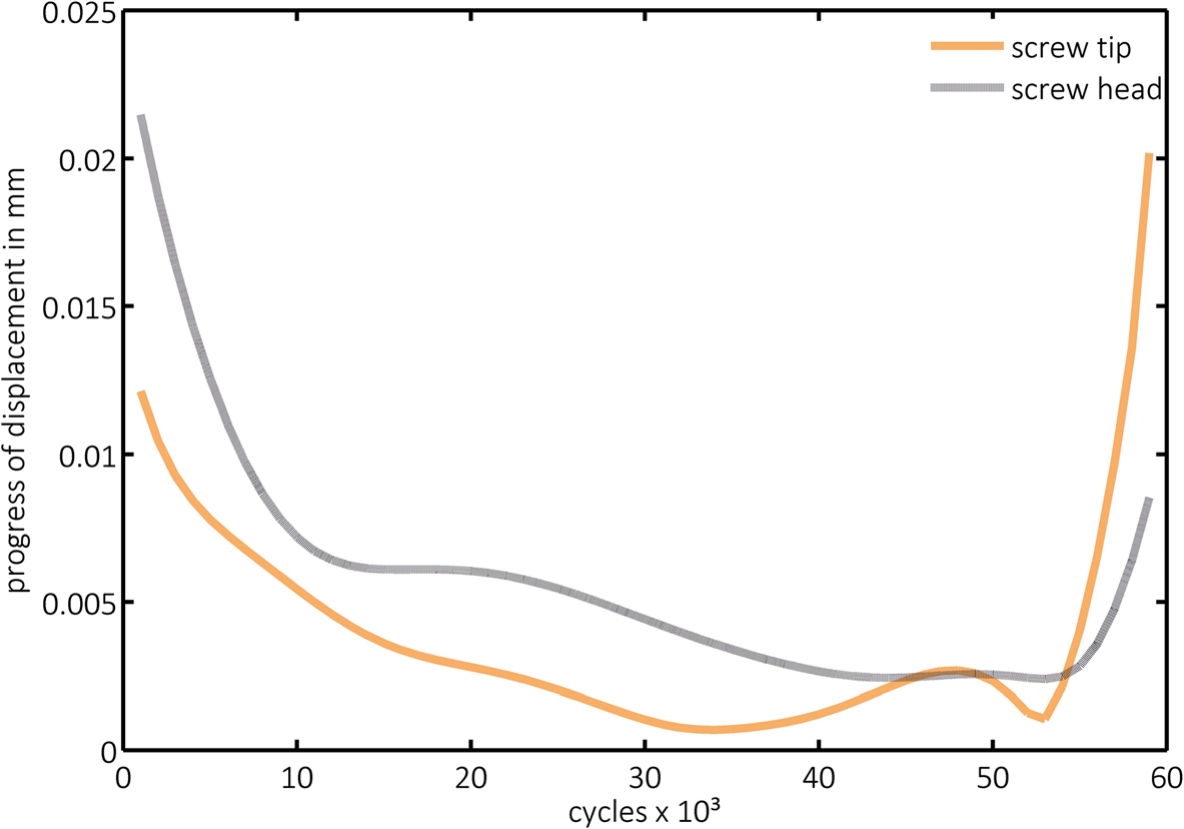
Exemplary progression of screw displacement in group 2 (augmented). The increase in progression toward the end of cyclic loading is obvious

**Fig. 8:**
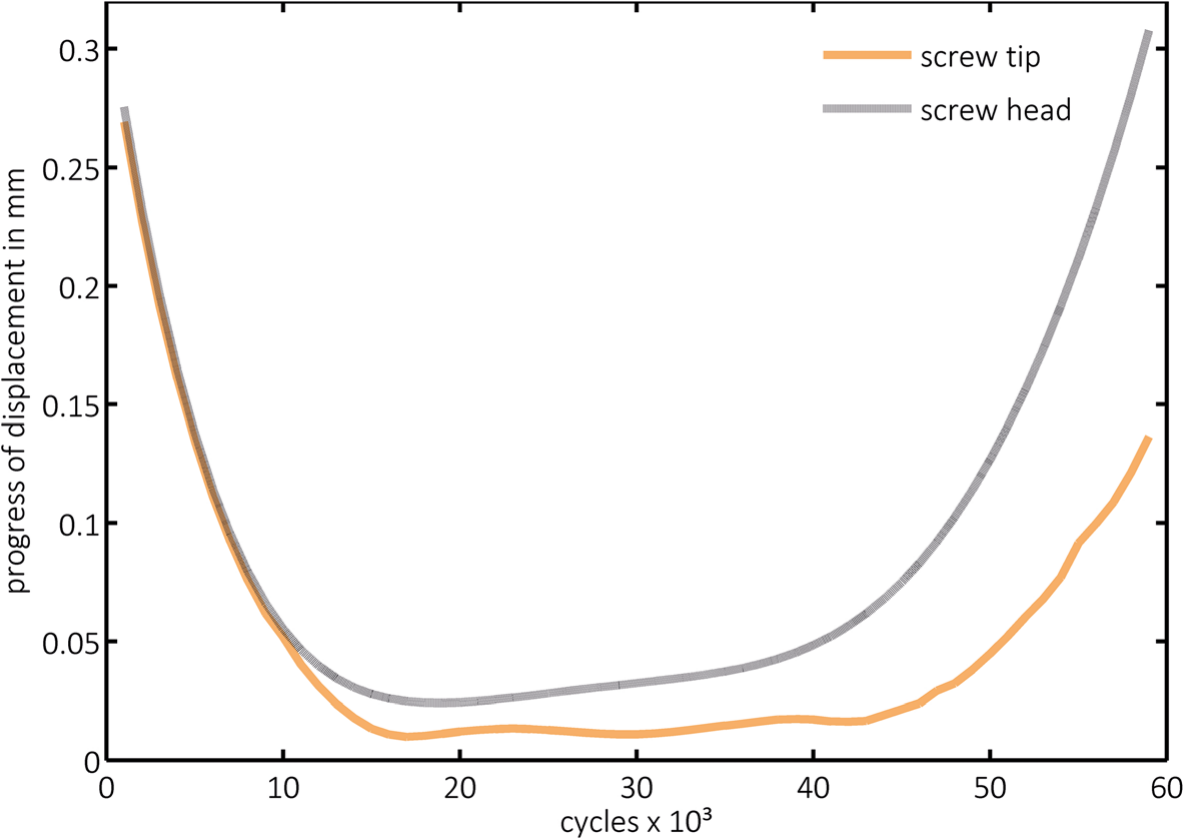
Progress of screw displacement of the abovementioned outlier in group 2 (augmented). The strong increase of the progression toward the end of the cyclic loading is remarkable

Progress of displacement (PD) was highest in both groups within the first 1000 cycles and significantly lower in group 2 for the head (*p* = 0.000) and for the tip (*p* = 0.001) than in group 1. In the following increments, a lower PD was observed, but it increased again considerably within the last measured increments in both groups (Fig. 6). However, this effect was more obvious in the graphical visualization of group 2, presenting the image of tub-like diagrams (Fig. 7), which became even more obvious when a structural weakening was suspected (Fig. 8).

### Screw movement pattern

Pure rotation or combined movement (toggling) was predominant in both groups. Rotation was detected in both groups in approximately 30% of samples (group 1: 31.5%; group 2: 30%). Toggling was slightly more detectable in the augmented group. In group 1 (non-augmented), toggling occurred in 29.2% of samples. In group 2, toggling was more obvious, affecting 35% of samples. Pure translation was seen in approximately 10% of samples in group 1 and 13% in group 2. Movements not assignable to any of these categories were characterized as undefined movement patterns.

The morphological parameters of the pedicle were examined for possible correlations with the displacement parameters of the screws. The results of the correlations in connection with the parameters of the cyclic loading are summarized in Table 2. The median pedicle height was 17.0 (15.0/19.1) (13.1–19.7) mm, the width was 10.5 (9.3/13.3) (6.0–20.5) mm and the angle was 15.7 (14.5/17.3) (9.8–21.4) °.

**Table 2:** Correlation of morphological parameters with cyclic loading parameters (*n* = 10)

Parameter		Group	Pedicle height	Pedicle width	Pedicle angle
**preload**	**Pdisp head**	1	0.455	0.758*	0.600
		2	0.462	0.186	− 0.373
	**Pdisp tip**	1	0.552	0.309	0.382
		2	0.144	0.344	0.354
**cyclic loading**	**Cdisp head**	1	−0.067	0.297	0.806**
		2	0.527	0.810*	0.048
	**Cdisp tip**	1	0.042	0.321	0.794**
		2	0.386	0.323	−0.072
	**Cmov headstart/end**	1	−0.049/0.317	0.286/0.585	0.480/0.476
		2	0.454/0.675	0.762*/0.790*	0.024/−0.060
	**Cmov tip start/end**	1	−0.552/− 0.140	– 0.212/− 0.097	0.309/− 0.152
		2	0.319/0.404	0.333/0.946**	0.024/−0.204
	**PD head**	1	0.321	0.285	0.721*
		2	0.590	0.515	−0.180
	**PD tip**	1	0.309	0.067	0.600
		2	−0.144	0.024	0.429

* significant (p < 0.05), ** highly significant (p < 0.01)

### Correlation to morphological parameters

In group 1, for the displacement under preload between condition 0 and condition 1, a correlation for pedicle width (*R* = 0.758) was demonstrated at the head. The pedicle angle correlated with screw displacement under cyclic loading at the head and tip (*R* = 0.806, *R* = 0.794).

In group 2, a correlation was found between the pedicle width and screw displacement under cyclic loading at the head (*R* = 0.810). In addition, in this group, the pedicle width correlated with screw movement at the head for the first and final cycles (*R* = 0.762, *R* = 0.790). However, a correlation with screw movement at the tip was only found for the final cycles (*R* = 0.946).

The pedicle angle in group 1 also correlated with PD at the head/tip (*R* = 0.721/*R* = 0.600), whereas in group 2, no correlations with PD could be found. For all other displacement or movement parameters, no correlation or only a weak correlation with other morphological parameters could be detected.

## Discussion

Interpreting primary stability at the bone-to-implant interface is an ongoing challenge in clinical routine and biomechanical research, and this particularly applies to osteopenic and osteoporotic vertebrae. While the enhancing effects of screw augmentation in a traditional pull-out setup have already been reported, the analysis of the loosening mechanisms in a comparison of left and right pedicles is more complex [1–9].

In the current study, the characteristics of pedicle screws were extensively analyzed based on the physiology and its cyclic load conditions to amplify known findings from classical pull-out tests [1, 6–8, 19–22]. The applied loads were consistent with in vivo loads [10]. The comparison of natively inserted and augmented screws revealed previously undescribed differences between the two procedures at the bone-to-implant interface under dynamic loading.

For each group, one sample showed a clear deviation from the other cyclic values. For one sample in the augmented group a medial perforation of the pedicle cortex was detected. Therefore, anchorage failure in the pedicle was suspected. This possibility was taken into account accordingly. In the non-augmented group one specimen had a macroscopic fracture in the pedicle base area. According to the findings of Newcomb et al., this bony area is exposed to particularly high stress [23]. Due to osteoporosis, the cortical bone in the pedicle is particularly thin and fragile [24]. Both samples were excluded from further statistical evaluation. The statistical analysis of the preloading was therefore performed with *n* = 12 and the statistical analysis of the cyclical loading with *n* = 10 screws for each group.

A standard instrumentation with a pedicle screw system consists bilaterally of at least one cranial and one caudal pedicle screw, each rigidly connected with a longitudinal rod. This condition should be considered when comparing the results with other studies. Various authors used a unilateral hinge or ball joint instead of a rigid connection between the rod and the machine actuator and expected to observe a craniocaudal loosening of the screws [8, 9, 25–28]. Consequently, the applied load did not simulate the situation of dorsal rigid instrumentation, limiting the comparison to the conclusions of the previous studies on primary stability at the bone-to-implant interface.

The preload on the screws in both groups showed only a low displacement with opposite behavior for the screw head and the screw tip in connection with a macroscopic deflection of the vertebra in extension. Augmentation reduced the displacement, but the reduction was only significant for the head. These observations are not necessarily to be interpreted as a loss of stability unless the bone-to-implant interface has a sufficient load-bearing capacity. This effect could possibly occur while first mobilizing the patient after surgery or even by reactivating trunk muscles. The initial displacement shown in our study does not necessarily result in early failure. It seems that the screws have to find an ideal fixed position under initial load application. In this context, osteoporosis may influence this mechanism, and it may be correlated with an early loosening effect, as the findings of Haines et al. (2013) describe during rod reduction by persuading the rod into the screw or performing a repositioning maneuver in osteoporosis [29].

The subsequent cyclic loading simulated a condition similar to that of the first postoperative weeks during walking. Displacement under cyclic loading showed 40% greater motion at the screw head than at the screw tip in group 1. With regard to the vertebral column, the screw head moved cranially, whereas the screw tip shifted caudally. This difference results from the degree of freedom of the setup, allowing a backward tilting of the vertebra. The initial screw alignment, e.g., parallel to the upper end plate of the vertebral body, no longer exists, and the screw toggles. By augmenting the screws, this effect could be reduced to less than one-fifth at the screw head and one-twelfth at the screw tip, thus almost maintaining the original screw position. Transferring this effect to the stiffness at the bone-to-implant interface, the augmentation led to an increase of 2.5–6-fold compared to the non-augmented condition. Additionally, there was a significant link of early screw failure and non-augmented screws.

The movement under cyclic loading demonstrated two key effects:

1. The screw movement at the tip was natively, e.g., without augmentation, 1.5-fold higher than at the head, which describes the clinically observed windshield wiper effect. At the screw tip, the thread has a small area for load distribution. This surface represents the contact surface to the surrounding trabeculae and is stressed both on compression and on shear. The shear strength of the trabecula is considerably lower than its compressive strength and thus represents the unfavorable load [30]. Under cyclic loading, the weak structures of the osteoporotic cancellous bone are compressed and sheared off until failure, which reflects their low load-bearing capacity [3]. An increasing screw movement results. In comparison, the augmentation of the screws in group 2 results in a strong expansion of the contact surface to the surrounding trabecular structure. The effect is a distribution of the load over a larger number of trabeculae, which reduces the load peaks of individual trabecula and, in turn, leads to a higher load-bearing capacity of the bone-to-implant interface. The simplified physical principle of surface pressure, which results from the quotient of force and surface, has a strong effect here. Accordingly, an increase in the surface area at constant force causes a reduction in the surface pressure.
2. The movement of head and tip decreased in the majority of screws in both groups, which may indicate a canting of the screw within the pedicle. This result corresponds to the displacement under cyclic loading, which showed a deflection of the vertebra toward extension. These effects and their combinations were observed for both left and right pedicles with side shifts between both during the load cycles (Fig. 5). In conclusion, instrumentation on both sides of the vertebra and its simultaneous loading represents an adequate way to investigate changes in load distribution and primary stability.

The progress of displacement was highest in both groups within the first cycles, decreased in the intermediate cycles, but increased again within the final cycles up to the abort criterion – defined as height reduction of 10 mm under preload and 15 mm under preload plus cyclic loading – or up to the total number of cycles of 60,000. This pattern might indicate that the yield strength of the cortical structures has been reached and that the pedicles may have failed.

In this context, one major limitation affecting all in vitro experiments at time is that physiological remodeling processes of the bone cannot be considered.

The analysis of the motion patterns was expected to include three essential patterns: translation, rotation, and the combination of both, which was defined as toggling. However, only a small percentage of pure translation was observed. In the test set-up, pure translation was possible if, for instance, two conditions were met: (i) the screw diameter and the pedicle dimensions did not match adequately and (ii) the trabeculae within the region of the screw tip had a low load capacity. As a result, the screw head and the screw tip can move in the same direction. This effect needs to be considered by reviewing findings from pure pull-out testing [1, 6–8, 20–22, 31].

**Fig. 9:**
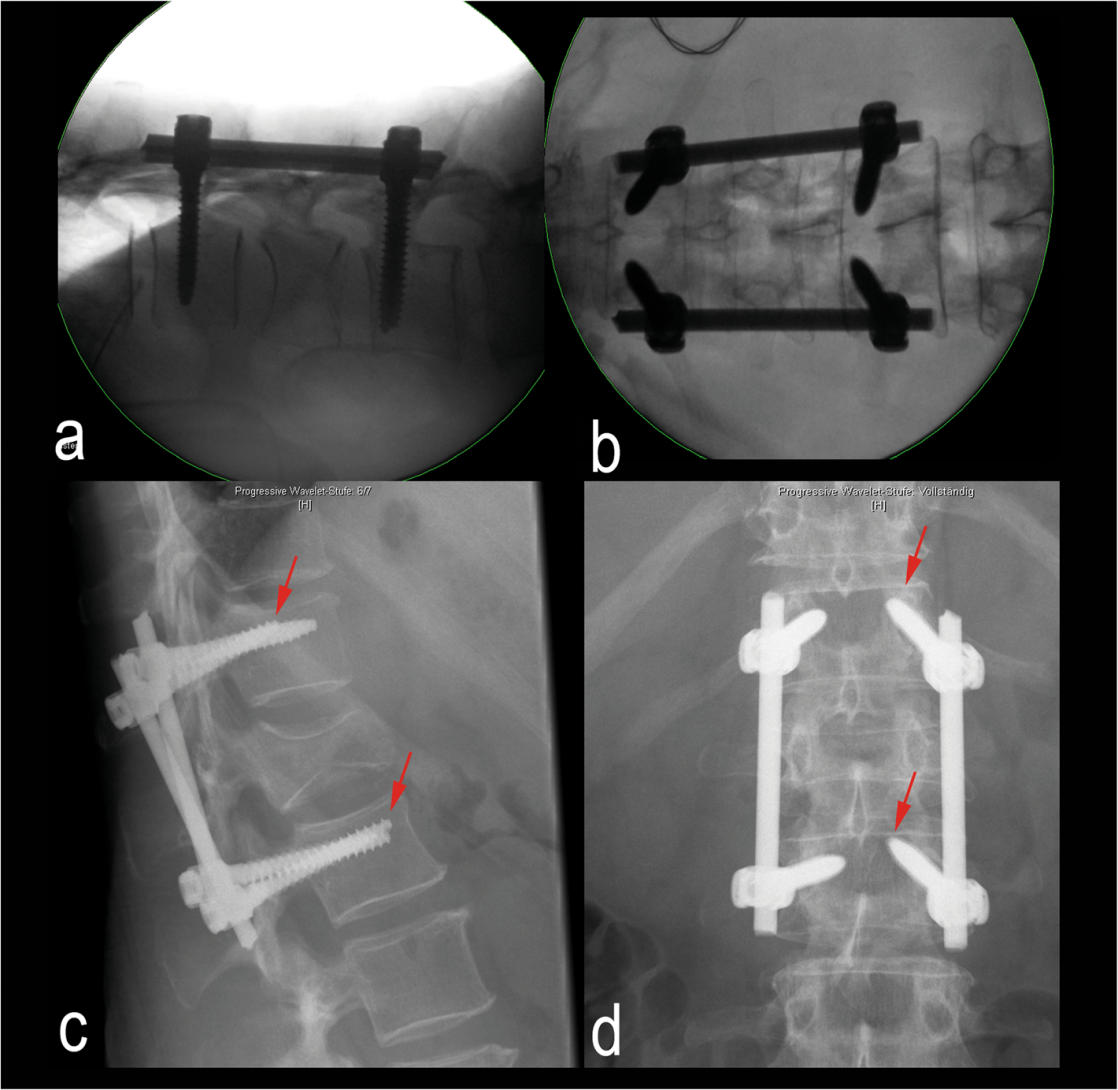
Intraoperative radiographs in prone position in lateral view (**a**) and ap view (**b**). Early implant failure 4 days after surgery in standing radiograph lateral view (**c**) and ap view (**d**) showing a combined movement (toggling) (red arrows)

Most of the movements were rotation and toggling. Rotation was possible under preload because the rigid connection between the screw head and the longitudinal fusion rod transferred the load to the bone-to-implant interface. This is the case, for instance, if the area near the screw head is in contact with the cortical bone of the pedicles and the screw. As a result, the screw tip can rotate around this pivot point, reflecting the typical clinically observed windshield wiper effect (Fig. 9).

The predominant movement pattern of rotation and toggling may explain the clinically observed failure pattern and may suggest a pathophysiological basis of screw failure (Fig. 2). Furthermore, the previously published beneficial effects achieved by using cross-connectors, index screws in short constructs or even long construct configuration could be explained by their capacity to reduce rotation [32, 33].

It is observed that cyclically, the extension of the movement at the screw tip undulates over the cycles (Fig. 5), which is probably because the trabeculae on the cement-to-implant interface form a more stable abutment shortly after failure due to compression, until this support is also weakened by the repetitive load, and the next trabeculae are damaged.

The morphological parameters were analyzed for a possible correlation with implant loosening. These parameters varied considerably between the two groups. While in group 1, a correlation between pedicle morphology and displacement under preload was found, no correlation could be found in group 2.

It is well known that an increase in screw size in relation to pedicle diameter will increase screw anchorage in the pedicle [34]. However, there is an increased risk of pedicle breakage by “oversizing” the screw. Hirano et al. found an increased fracture risk by screws > 70% of the outer diameter of the pedicle in osteoporosis [35]. By augmenting the screw, this balancing act of finding the right screw size depending on pedicle size might be become less critical for early screw movement.

The pedicle angle and displacement under cyclic loading in group 1 were also correlated. This result suggests that a larger pedicle angle leads to an increased cyclical displacement, i.e., cyclical subsidence behavior.

This factor needs to be discussed by taking into account that we exclusively used 45 mm screws. With an increased pedicle angle, the screws were shorter in relation to the vertebral body. Therefore, we did not take advantage of the possibility of using longer screws by increasing the insertion angle. However, screw length and depth of insertion are other known important factors of screw anchorage [36].

Increasing the insertion angle in our setup enabled the screw tip to be placed more centrally in the vertebral body. One reason for the correlation between pedicle angle and cyclical displacement could be an inhomogeneous distribution of the trabecular bone structure within the vertebral body [37]. With an increasing pedicle angle, the converging screw tips are positioned in an area of sparse trabecular structure near the vertebral body center and cyclical displacement can increase. As the angle increases, the screws continue to converge and stability decreases. The effects of converging and diverging screws were previously described in an osteoporotic bone model [38, 39]. A recent study by Newcomb et al. investigated the effects of different pedicle screw trajectories on stresses in cortical and cancellous bone using finite element methods [23]. They found that under simulated flexion movement, the medial trajectory corresponding to the converging axes of the left and right pedicle canals caused the greatest stress to the cancellous bone. This effect provides further evidence for the present relationship, indicating that cyclical displacement increases with the pedicle angle in our model.

A relationship between the pedicle angle and the cyclical displacement could not be observed in group 2. As described above, an increase in the contact surface for load transfer was achieved by the bone cement. It is therefore reasonable to assume that the linear relationship between pedicle angle and cyclical displacement disappears. These findings may contribute to our understanding of the mechanisms of cement augmentation, screw placement and its correspondent individual morphometric relation to an osteoporotic vertebra.

**Fig. 10:**
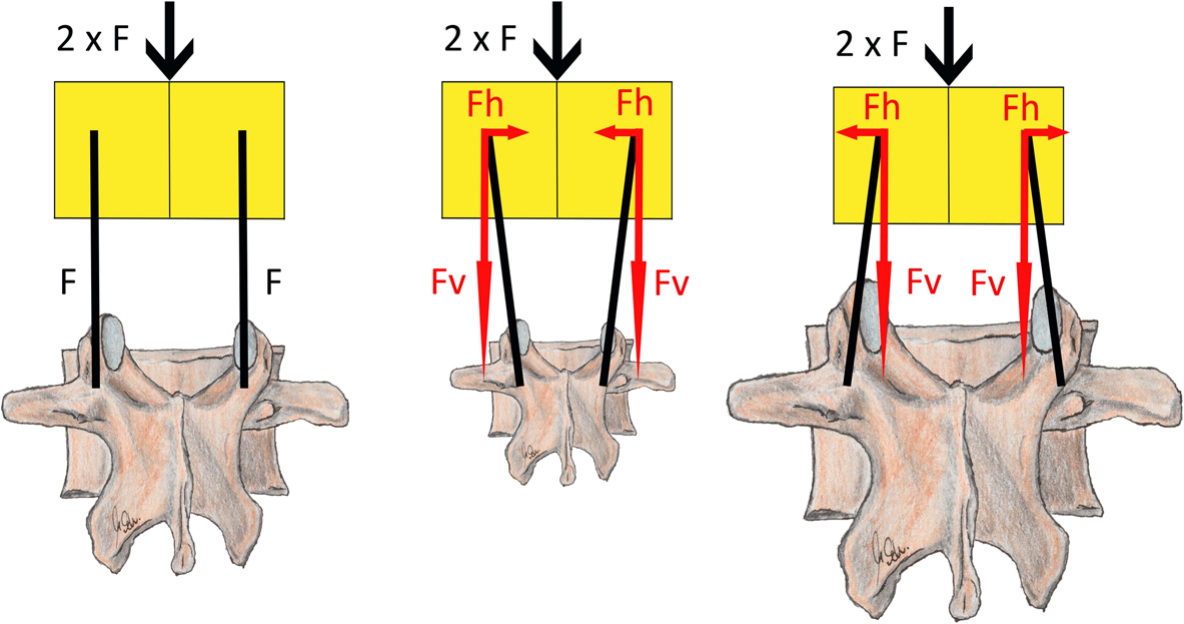
Change of the direction of the screw load depending on the alignment of the connecting rods (black) - on the left side the vertical force component corresponds to the perpendicular (F). As the rods are aligned out of the perpendicular (F), the vertical applied load also acts in the horizontal (Fh) and the vertical force component (Fv) decreases; shown in the middle and on the right: pedicles that are more narrowly and more widely spaced

A correlation was found between the pedicle width and the cyclical displacement at the screw head. Thus, an increase in cyclic subsidence would also be expected with increasing pedicle width. A look at the physics of the pedicle screw system is useful here (see Fig. 10). The resulting force vectors, which transfer the axial load from the cranial to the caudal vertebra, depend on the alignment of the longitudinal dorsal connecting rods. The resulting force vector can easily be divided into its components in vertical and horizontal directions. As the horizontal component increases, the lateral forces increase on the pedicles. In general, however, the ratio of pedicle width and pedicle height is more in favor of height and the vertical force vector contributes the main component of the sum vector. A correlation between pedicle height and cyclical displacement would be expected. The same consideration applies to the interpretation of the existing correlations of pedicle width and cyclical movement. Instead of the pedicle width, a relationship between the pedicle height and the cyclical movement would have been more likely.

The importance of adequate screw dimensions in correlation with the individual anatomy of each vertebra has long been recognized [36].

Kiner et al. showed more than 10 years ago that the diameter of the screw is more important for stability than the augmentation of a screw with a smaller diameter, and consequently, the pedicle dimension and screw diameter should be well matched [39].

The present results not only confirm that the combination of pedicle dimension, screw diameter and augmentation have a decisive influence on primary stability but also contribute new findings on consecutive complex screw movement.

To our knowledge, this is the first study to describe complex screw movement with and without augmentation in an osteoporotic spine model. The presented findings may contribute to a better understanding of screw anchorage and potential failure mechanisms.

## Conclusions

The cyclic loading based on physiological conditions during walking has allowed the postoperative conditions and clinical failure mechanisms to be simulated in vitro. The interaction between the left and right screws on the bone-to-implant interface that were demonstrated here improves our understanding of the failure mechanisms. This information offers new possibilities for the development and evaluation of specific treatment options for the osteoporotic spine.

The optically measured movements of the screws allow indirect conclusions on the complex load distribution and its changes under axial cyclic loading. Thus, it could be shown that the failure between the left and right sides does not occur simultaneously to the same extent; rather, it occurs successively and depends on a possible abutment within the pedicle. Most of the subsidence took place at the beginning of the cyclic loading. In this context, early primary stability can provide an indication of overall stability. It was also shown that augmentation of the screws in poor bone quality reduces screw subsidence, which improves the rigidity of the screw-to-implant interface by up to six-fold. The non-augmented condition was significantly related to early screw failure. In addition to the movement parameters, different movement categories were determined, and both native and augmented screws are subject to a similar distribution. An analysis of the change in movement patterns over time would also be interesting in the future.

## Data Availability

The raw data of all measurements and imaging represent a large amount of data and are subject to further analysis. They can therefore not yet be made available as a supplement to this manuscript.

## Abbreviations

ASTM: American society of materials and testing
BMD: Bone mineral density.
Cdisp: Subsidence under cyclic loading.
DEFL: Total deflection.
PD: Progress of displacement.
Pdefl: Deflection under preload.
Pdisp: Subsidence under preload.
PDmax: Maximum progress of displacement.
PEEK: Organic thermoplastic polymer.
PMMA: Polymethyl methacrylate cement.
Pstiff: Stiffness under preload.
STIFF: Total stiffness, Cmov: screw loosening.
